# (*E*)-2-[1-(3-Amino-4-chloro­phenyl­imino)eth­yl]-4-bromo­phenol

**DOI:** 10.1107/S1600536810009773

**Published:** 2010-03-20

**Authors:** Hadariah Bahron, Siti Najihah Abu Bakar, Karimah Kassim, Chin Sing Yeap, Hoong-Kun Fun

**Affiliations:** a Faculty of Applied Sciences, Universiti Teknologi MARA, 40450, Shah Alam, Malaysia; b X-ray Crystallography Unit, School of Physics, Universiti Sains Malaysia, 11800 USM, Penang, Malaysia

## Abstract

The title Schiff base compound, C_14_H_12_BrClN_2_O, exists in an *E* configuration with respect to the central C=N double bond. The amino group adopts a pyramidal configuration. The dihedral angle between the two benzene rings is 76.88 (10)° and an intra­molecular O—H⋯N hydrogen bond forms a six-membered ring, generating an *S*(6) ring motif. In the crystal structure, mol­ecules are linked into chains along [010] *via* N—H⋯O hydrogen bonds. The presence of π–π inter­actions [centroid–centroid distance = 3.6244 (12) Å] further stabilizes the crystal structure.

## Related literature

For the biological activity and corrosion inhibition properties of Schiff base derivatives, see: Azam *et al.* (2007 ▶); Sauri *et al.* (2009 ▶). For a related structure, see: Yamin *et al.* (2009 ▶). For hydrogen-bond motifs, see: Bernstein *et al.* (1995 ▶).
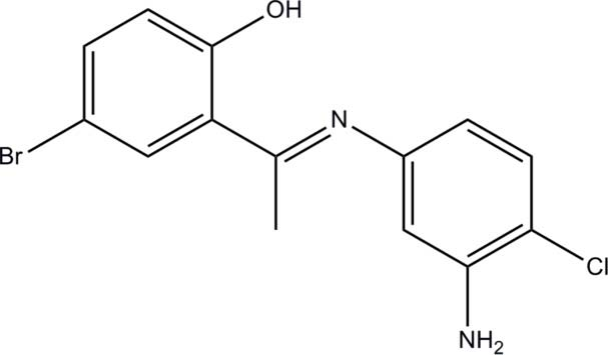


## Experimental

### 

#### Crystal data

C_14_H_12_BrClN_2_O
*M* *_r_* = 339.62
Monoclinic, 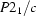

*a* = 10.2469 (1) Å
*b* = 8.7672 (1) Å
*c* = 15.7180 (2) Å
β = 107.065 (1)°
*V* = 1349.88 (3) Å^3^
*Z* = 4
Mo *K*α radiation
μ = 3.24 mm^−1^
*T* = 296 K
0.24 × 0.22 × 0.11 mm

#### Data collection

Bruker SMART APEXII CCD area-detector diffractometer
Absorption correction: multi-scan (*SADABS*; Bruker, 2009 ▶) *T*_min_ = 0.509, *T*_max_ = 0.726
14782 measured reflections
3925 independent reflections
2420 reflections with *I* > 2σ(*I*)
*R*_int_ = 0.029

#### Refinement

*R*[*F* ^2^ > 2σ(*F* ^2^)] = 0.035
*wR*(*F* ^2^) = 0.077
*S* = 1.00
3925 reflections
185 parameters
H atoms treated by a mixture of independent and constrained refinement
Δρ_max_ = 0.28 e Å^−3^
Δρ_min_ = −0.27 e Å^−3^

### 

Data collection: *APEX2* (Bruker, 2009 ▶); cell refinement: *SAINT* (Bruker, 2009 ▶); data reduction: *SAINT*; program(s) used to solve structure: *SHELXTL* (Sheldrick, 2008 ▶); program(s) used to refine structure: *SHELXTL*; molecular graphics: *SHELXTL*; software used to prepare material for publication: *SHELXTL* and *PLATON* (Spek, 2009 ▶).

## Supplementary Material

Crystal structure: contains datablocks global, I. DOI: 10.1107/S1600536810009773/tk2641sup1.cif

Structure factors: contains datablocks I. DOI: 10.1107/S1600536810009773/tk2641Isup2.hkl

Additional supplementary materials:  crystallographic information; 3D view; checkCIF report

## Figures and Tables

**Table 1:** Hydrogen-bond geometry (Å, °)

*D*—H⋯*A*	*D*—H	H⋯*A*	*D*⋯*A*	*D*—H⋯*A*
O1—H1*O*1⋯N1	0.86 (3)	1.74 (3)	2.533 (2)	151 (3)
N2—H1*N*2⋯O1^i^	0.82 (3)	2.33 (3)	3.129 (3)	163 (2)

Symmetry code: (i) 
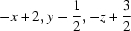
.

**Fig. 1.**
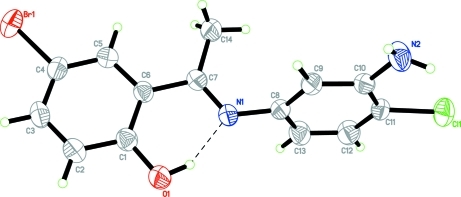
The molecular structure of (I) with 50% probability ellipsoids for non-H atoms. An intramolecular hydrogen bond is shown as a dashed line.

**Fig. 2.**
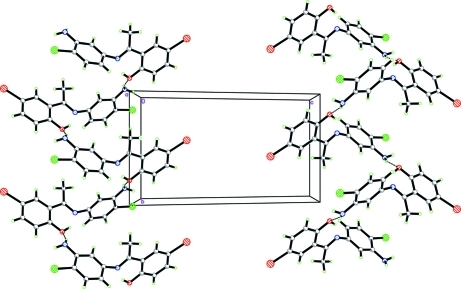
A view down the a axis of the unit cell of (I) showing molecules linked into one-dimensional chains along [010]. Intermolecular hydrogen bonds are shown as dashed lines.

## supplementary crystallographic information

### Comment

Schiff bases have been studied extensively due to their intriguing biological activities, such as antimicrobial (Azam *et al.*, 2007), and chemical properties as well as corrosion inhibition (Sauri *et al.*, 2009). The structure of a Schiff base synthesized from 1,3-diamino-4-chlorobenzene and 3-methoxysalicylaldehyde in 1:2 ratio has been reported by Yamin *et al.* (2009). The present Schiff base compound, (I), is also derived from 1,3-diamino-4-chlorobenzene but from an analogous reaction with 5-bromo-2-hydroxyacetophenone.

Compound, (I), exists in an *E* configuration with respect to the central C7═N1 double bond (Fig. 1). The dihedral angle between the two benzene rings is 76.88 (10)°. The amino group (N2) adopts a pyramidal configuration. An intramolecular O1—H1O1···N1 hydrogen bond forms a six-membered ring, generating an S(6) ring motif (Bernstein *et al.*, 1995). In the crystal structure, the molecules are linked into one-dimensional chains along [010] *via* intermolecular N2—H1N2···O1 hydrogen bonds (Fig. 2, Table 1). The Cg1···Cg2 interaction of 3.6244 (12) Å; x, 5/2-y, 1/2+z, further stabilizes the crystal structure (Cg1 and Cg2 are centroids of benzene rings C8–C13 and C1–C6, respectively).

### Experimental

Compound (I) was synthesized by heating 1,3-diamino-4-chlorobenzene (0.3565 g, 2.5 mmol) with 5-bromo-2-hydroxyacetophenone (0.998 g, 5 mmol) in ethanol for 24 h. The solvent was then evaporated in-*vacuo* and the oily product was recrystallized from acetone to afford yellow single crystals. Yield 12%. Melting point 448-452 K.

### Refinement

The H1O1, H1N2 and H2N2 hydrogen atoms were located from a difference Fourier map and refined freely. The remaining H atoms were positioned geometrically and refined using a riding model, with C–H = 0.93 or 0.96 Å, and with *U*_iso_(H) = 1.2 or 1.5 *U*_eq_(C). The rotating group model was applied for the methyl groups.

### Crystal data

**Table:**

C_14_H_12_BrClN_2_O	*F*(000) = 680
*M**_r_* = 339.62	*D*_x_ = 1.671 Mg m^−^^3^
Monoclinic, *P*2_1_/*c*	Mo *K*α radiation, λ = 0.71073 Å
Hall symbol: -P 2ybc	Cell parameters from 4343 reflections
*a* = 10.2469 (1) Å	θ = 2.7–28.5°
*b* = 8.7672 (1) Å	µ = 3.24 mm^−^^1^
*c* = 15.7180 (2) Å	*T* = 296 K
β = 107.065 (1)°	Block, yellow
*V* = 1349.88 (3) Å^3^	0.24 × 0.22 × 0.11 mm
*Z* = 4	

### Data collection

**Table:**

Bruker SMART APEXII CCD area-detector diffractometer	3925 independent reflections
Radiation source: fine-focus sealed tube	2420 reflections with *I* > 2σ(*I*)
graphite	*R*_int_ = 0.029
φ and ω scans	θ_max_ = 30.1°, θ_min_ = 2.1°
Absorption correction: multi-scan (*SADABS*; Bruker, 2009)	*h* = −14→14
*T*_min_ = 0.509, *T*_max_ = 0.726	*k* = −12→9
14782 measured reflections	*l* = −22→21

### Refinement

**Table:**

Refinement on *F*^2^	Primary atom site location: structure-invariant direct methods
Least-squares matrix: full	Secondary atom site location: difference Fourier map
*R*[*F*^2^ > 2σ(*F*^2^)] = 0.035	Hydrogen site location: inferred from neighbouring sites
*wR*(*F*^2^) = 0.077	H atoms treated by a mixture of independent and constrained refinement
*S* = 1.00	*w* = 1/[σ^2^(*F*_o_^2^) + (0.0299*P*)^2^ + 0.2742*P*] where *P* = (*F*_o_^2^ + 2*F*_c_^2^)/3
3925 reflections	(Δ/σ)_max_ = 0.001
185 parameters	Δρ_max_ = 0.28 e Å^−^^3^
0 restraints	Δρ_min_ = −0.27 e Å^−^^3^

### Special details

**Table:**

Geometry. All esds (except the esd in the dihedral angle between two l.s. planes) are estimated using the full covariance matrix. The cell esds are taken into account individually in the estimation of esds in distances, angles and torsion angles; correlations between esds in cell parameters are only used when they are defined by crystal symmetry. An approximate (isotropic) treatment of cell esds is used for estimating esds involving l.s. planes.
Refinement. Refinement of *F*^2^ against ALL reflections. The weighted *R*-factor wR and goodness of fit *S* are based on *F*^2^, conventional *R*-factors *R* are based on *F*, with *F* set to zero for negative *F*^2^. The threshold expression of *F*^2^ > σ(*F*^2^) is used only for calculating *R*-factors(gt) etc. and is not relevant to the choice of reflections for refinement. *R*-factors based on *F*^2^ are statistically about twice as large as those based on *F*, and *R*- factors based on ALL data will be even larger.

### Fractional atomic coordinates and isotropic or equivalent isotropic displacement parameters (Å^2^)

**Table:**

	*x*	*y*	*z*	*U*_iso_*/*U*_eq_	
Br1	0.82408 (3)	0.92283 (3)	0.233924 (15)	0.05982 (11)	
Cl1	0.55171 (6)	1.10458 (7)	0.92352 (4)	0.05352 (17)	
O1	0.92270 (16)	1.31628 (18)	0.55948 (10)	0.0499 (4)	
N1	0.76555 (17)	1.1434 (2)	0.61354 (10)	0.0391 (4)	
N2	0.7688 (2)	0.9064 (2)	0.89348 (14)	0.0503 (5)	
C1	0.9012 (2)	1.2211 (2)	0.48934 (12)	0.0361 (5)	
C2	0.9613 (2)	1.2577 (3)	0.42328 (13)	0.0415 (5)	
H2A	1.0168	1.3435	0.4298	0.050*	
C3	0.9398 (2)	1.1686 (3)	0.34850 (13)	0.0411 (5)	
H3A	0.9795	1.1944	0.3043	0.049*	
C4	0.8588 (2)	1.0407 (2)	0.33972 (12)	0.0376 (5)	
C5	0.7997 (2)	1.0004 (2)	0.40428 (13)	0.0377 (5)	
H5A	0.7462	0.9130	0.3971	0.045*	
C6	0.81908 (19)	1.0898 (2)	0.48087 (12)	0.0325 (4)	
C7	0.75211 (19)	1.0490 (2)	0.54920 (13)	0.0341 (5)	
C8	0.7052 (2)	1.1213 (2)	0.68396 (12)	0.0349 (5)	
C9	0.7605 (2)	1.0181 (2)	0.75146 (13)	0.0361 (5)	
H9A	0.8302	0.9534	0.7471	0.043*	
C10	0.71348 (19)	1.0095 (2)	0.82588 (12)	0.0343 (5)	
C11	0.6078 (2)	1.1068 (2)	0.82884 (13)	0.0353 (5)	
C12	0.5507 (2)	1.2078 (3)	0.76140 (14)	0.0457 (5)	
H12A	0.4790	1.2703	0.7648	0.055*	
C13	0.6000 (2)	1.2166 (3)	0.68838 (14)	0.0442 (5)	
H13A	0.5626	1.2857	0.6429	0.053*	
C14	0.6714 (2)	0.9042 (2)	0.53932 (15)	0.0497 (6)	
H14A	0.6292	0.8973	0.5862	0.075*	
H14B	0.7311	0.8186	0.5426	0.075*	
H14C	0.6022	0.9040	0.4828	0.075*	
H1O1	0.875 (3)	1.280 (3)	0.5918 (17)	0.090 (10)*	
H1N2	0.845 (3)	0.875 (3)	0.8945 (15)	0.056 (8)*	
H2N2	0.760 (3)	0.933 (3)	0.9416 (18)	0.068 (9)*	

### Atomic displacement parameters (Å^2^)

**Table:**

	*U*^11^	*U*^22^	*U*^33^	*U*^12^	*U*^13^	*U*^23^
Br1	0.0789 (2)	0.0630 (2)	0.04600 (15)	0.00123 (13)	0.03142 (13)	−0.01291 (12)
Cl1	0.0612 (4)	0.0630 (4)	0.0472 (3)	0.0015 (3)	0.0328 (3)	−0.0020 (3)
O1	0.0632 (10)	0.0497 (10)	0.0423 (9)	−0.0224 (8)	0.0241 (8)	−0.0097 (7)
N1	0.0511 (11)	0.0378 (10)	0.0321 (9)	−0.0088 (8)	0.0180 (8)	−0.0014 (8)
N2	0.0585 (14)	0.0534 (14)	0.0435 (12)	0.0162 (11)	0.0222 (11)	0.0160 (10)
C1	0.0383 (11)	0.0381 (13)	0.0314 (10)	−0.0005 (9)	0.0095 (9)	0.0032 (9)
C2	0.0408 (12)	0.0428 (13)	0.0425 (12)	−0.0045 (10)	0.0149 (10)	0.0046 (10)
C3	0.0418 (12)	0.0469 (14)	0.0400 (11)	0.0060 (11)	0.0206 (10)	0.0106 (10)
C4	0.0426 (11)	0.0396 (13)	0.0320 (10)	0.0090 (10)	0.0131 (9)	0.0022 (9)
C5	0.0435 (12)	0.0327 (12)	0.0391 (11)	0.0000 (10)	0.0157 (10)	0.0005 (9)
C6	0.0348 (10)	0.0327 (12)	0.0306 (10)	0.0014 (9)	0.0106 (8)	0.0037 (9)
C7	0.0378 (11)	0.0323 (12)	0.0328 (10)	−0.0004 (9)	0.0113 (9)	0.0033 (9)
C8	0.0420 (11)	0.0335 (12)	0.0303 (10)	−0.0072 (9)	0.0123 (9)	−0.0034 (9)
C9	0.0398 (11)	0.0340 (12)	0.0381 (11)	0.0030 (9)	0.0169 (9)	−0.0008 (9)
C10	0.0388 (11)	0.0318 (12)	0.0317 (10)	−0.0037 (9)	0.0094 (9)	0.0002 (9)
C11	0.0401 (11)	0.0370 (12)	0.0329 (10)	−0.0029 (9)	0.0173 (9)	−0.0036 (9)
C12	0.0457 (12)	0.0461 (14)	0.0489 (13)	0.0087 (11)	0.0195 (11)	0.0025 (11)
C13	0.0499 (13)	0.0445 (14)	0.0377 (11)	0.0061 (11)	0.0121 (10)	0.0082 (10)
C14	0.0631 (15)	0.0461 (15)	0.0480 (13)	−0.0175 (11)	0.0288 (11)	−0.0101 (11)

### Geometric parameters (Å, °)

**Table:**

Br1—C4	1.900 (2)	C5—C6	1.401 (3)
Cl1—C11	1.7458 (18)	C5—H5A	0.9300
O1—C1	1.348 (2)	C6—C7	1.478 (2)
O1—H1O1	0.86 (3)	C7—C14	1.498 (3)
N1—C7	1.282 (2)	C8—C13	1.382 (3)
N1—C8	1.431 (2)	C8—C9	1.383 (3)
N2—C10	1.384 (3)	C9—C10	1.392 (2)
N2—H1N2	0.82 (3)	C9—H9A	0.9300
N2—H2N2	0.82 (3)	C10—C11	1.390 (3)
C1—C2	1.392 (3)	C11—C12	1.373 (3)
C1—C6	1.409 (3)	C12—C13	1.385 (3)
C2—C3	1.374 (3)	C12—H12A	0.9300
C2—H2A	0.9300	C13—H13A	0.9300
C3—C4	1.378 (3)	C14—H14A	0.9600
C3—H3A	0.9300	C14—H14B	0.9600
C4—C5	1.372 (3)	C14—H14C	0.9600
			
C1—O1—H1O1	105.6 (19)	C6—C7—C14	119.31 (17)
C7—N1—C8	123.50 (17)	C13—C8—C9	120.47 (17)
C10—N2—H1N2	114.0 (17)	C13—C8—N1	118.56 (18)
C10—N2—H2N2	112.9 (18)	C9—C8—N1	120.61 (18)
H1N2—N2—H2N2	116 (2)	C8—C9—C10	120.98 (18)
O1—C1—C2	117.72 (19)	C8—C9—H9A	119.5
O1—C1—C6	122.22 (17)	C10—C9—H9A	119.5
C2—C1—C6	120.05 (19)	N2—C10—C11	121.52 (18)
C3—C2—C1	120.8 (2)	N2—C10—C9	121.06 (19)
C3—C2—H2A	119.6	C11—C10—C9	117.42 (18)
C1—C2—H2A	119.6	C12—C11—C10	121.97 (17)
C2—C3—C4	119.29 (18)	C12—C11—Cl1	119.54 (15)
C2—C3—H3A	120.4	C10—C11—Cl1	118.44 (15)
C4—C3—H3A	120.4	C11—C12—C13	119.95 (19)
C5—C4—C3	121.26 (19)	C11—C12—H12A	120.0
C5—C4—Br1	119.84 (16)	C13—C12—H12A	120.0
C3—C4—Br1	118.87 (14)	C8—C13—C12	119.2 (2)
C4—C5—C6	120.66 (19)	C8—C13—H13A	120.4
C4—C5—H5A	119.7	C12—C13—H13A	120.4
C6—C5—H5A	119.7	C7—C14—H14A	109.5
C5—C6—C1	117.94 (17)	C7—C14—H14B	109.5
C5—C6—C7	120.68 (18)	H14A—C14—H14B	109.5
C1—C6—C7	121.36 (17)	C7—C14—H14C	109.5
N1—C7—C6	116.84 (17)	H14A—C14—H14C	109.5
N1—C7—C14	123.84 (17)	H14B—C14—H14C	109.5
			
O1—C1—C2—C3	−177.75 (18)	C5—C6—C7—C14	4.6 (3)
C6—C1—C2—C3	1.2 (3)	C1—C6—C7—C14	−177.06 (19)
C1—C2—C3—C4	−0.8 (3)	C7—N1—C8—C13	−110.9 (2)
C2—C3—C4—C5	−0.2 (3)	C7—N1—C8—C9	76.0 (3)
C2—C3—C4—Br1	177.57 (15)	C13—C8—C9—C10	−1.2 (3)
C3—C4—C5—C6	0.6 (3)	N1—C8—C9—C10	171.82 (18)
Br1—C4—C5—C6	−177.08 (14)	C8—C9—C10—N2	−180.0 (2)
C4—C5—C6—C1	−0.2 (3)	C8—C9—C10—C11	1.0 (3)
C4—C5—C6—C7	178.20 (18)	N2—C10—C11—C12	−178.9 (2)
O1—C1—C6—C5	178.19 (18)	C9—C10—C11—C12	0.2 (3)
C2—C1—C6—C5	−0.7 (3)	N2—C10—C11—Cl1	3.7 (3)
O1—C1—C6—C7	−0.2 (3)	C9—C10—C11—Cl1	−177.24 (15)
C2—C1—C6—C7	−179.09 (18)	C10—C11—C12—C13	−1.1 (3)
C8—N1—C7—C6	179.02 (17)	Cl1—C11—C12—C13	176.27 (17)
C8—N1—C7—C14	0.2 (3)	C9—C8—C13—C12	0.2 (3)
C5—C6—C7—N1	−174.25 (18)	N1—C8—C13—C12	−172.91 (19)
C1—C6—C7—N1	4.1 (3)	C11—C12—C13—C8	0.9 (3)

### Hydrogen-bond geometry (Å, °)

**Table:**

*D*—H···*A*	*D*—H	H···*A*	*D*···*A*	*D*—H···*A*
O1—H1O1···N1	0.86 (3)	1.74 (3)	2.533 (2)	151 (3)
N2—H1N2···O1^i^	0.82 (3)	2.33 (3)	3.129 (3)	163 (2)

Symmetry codes: (i) −*x*+2, *y*−1/2, −*z*+3/2.
